# Supplementary figures and images for: Diatom abundance in the polar oceans is predicted by genome size
Source: PLoS Biol. 2024 Aug 8;22(8):e3002733. doi: 10.1371/journal.pbio.3002733 (PMC11309476; doi:10.1371/journal.pbio.3002733)

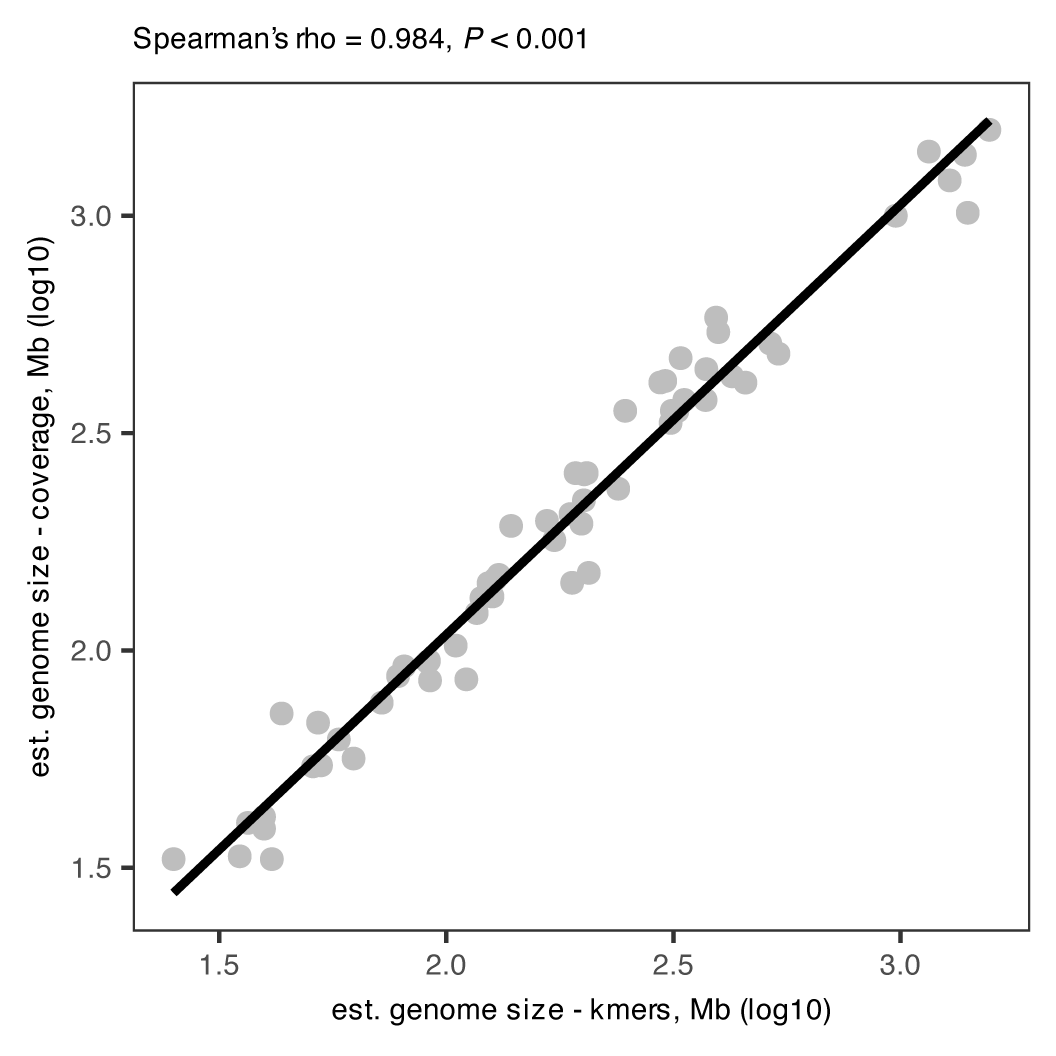

Supplement: S1 Fig — Scatterplot showing the correlation between estimated haploid genome sizes via k-mer- and coverage-based approaches. The black line indicates the regression coefficient. Spearman’s rho and associated P value are shown above the plot. The data and code to generate this figure can be found in https://doi.org/10.5281/zenodo.12608914. (TIF) [file pbio.3002733.s001.tif]

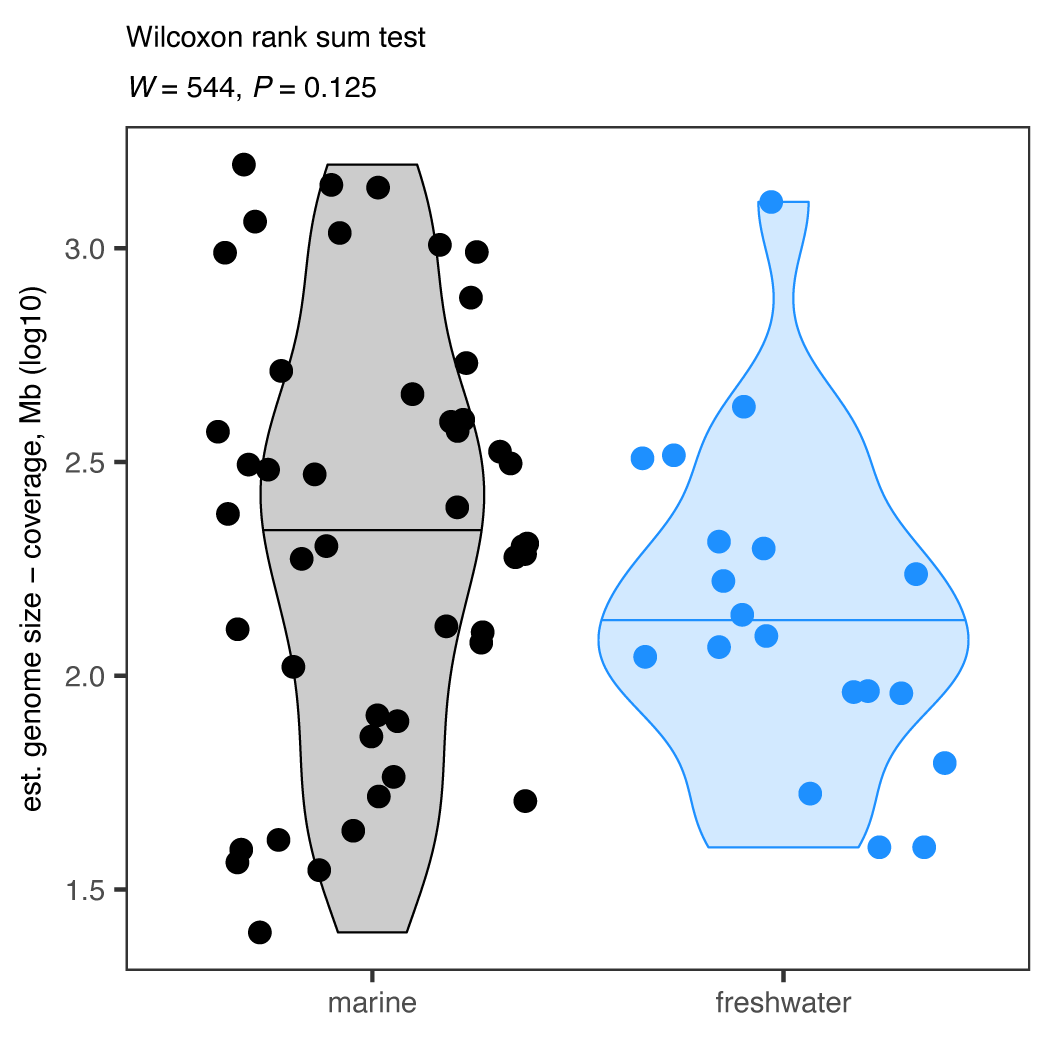

Supplement: S2 Fig — Violin plots showing the distribution of coverage-based haploid genome size estimates for marine (black) and freshwater (blue) species. The test statistic and P value from a Wilcoxon rank sum test are shown above the plot. The data and code to generate this figure can be found in https://doi.org/10.5281/zenodo.12608914. (TIF) [file pbio.3002733.s002.tif]

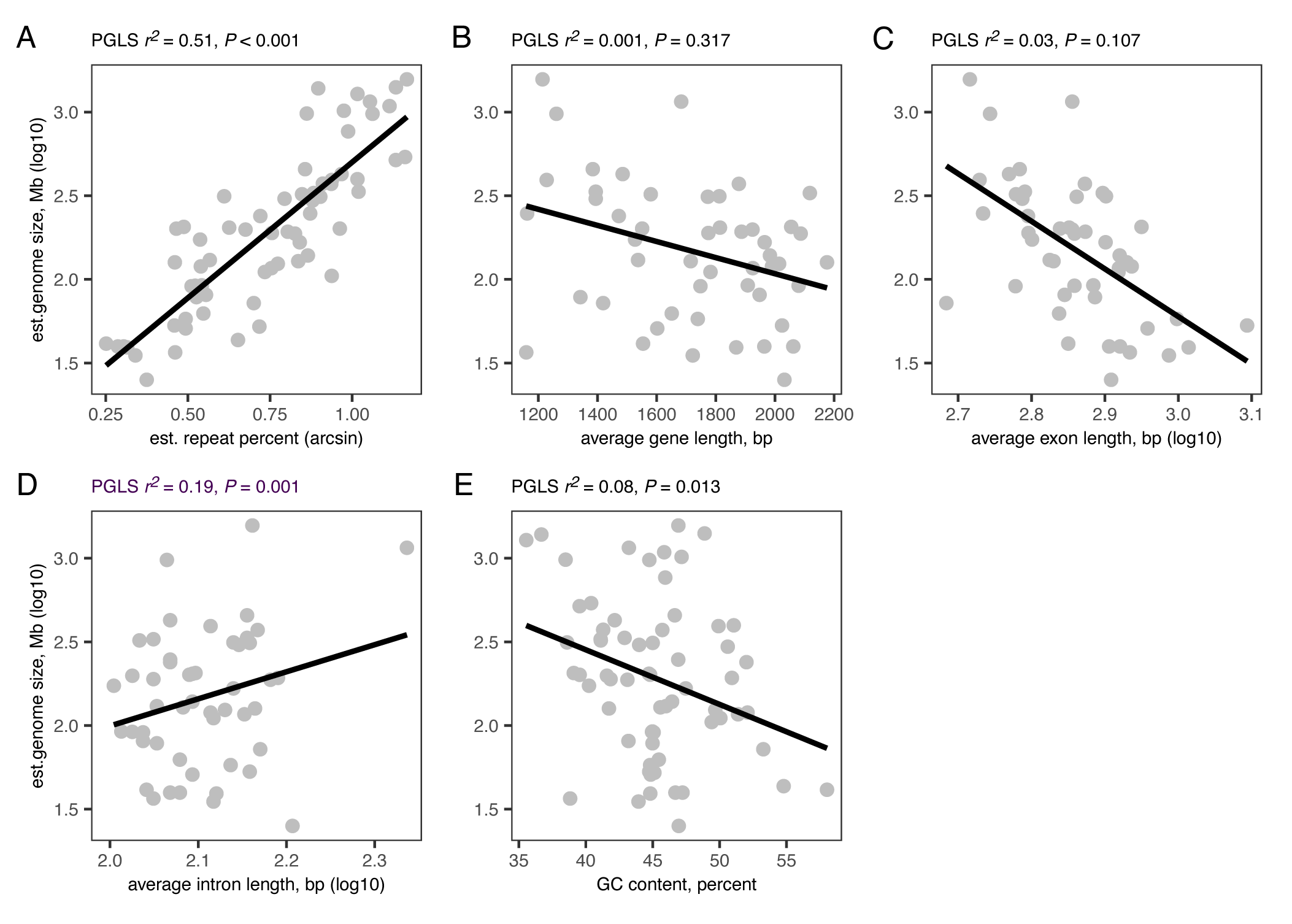

Supplement: S3 Fig — Phylogenetic generalized least squares (PGLS) models predicting genome size by (A) the estimated percentage of repetitive elements in the genome, (B) the average gene length, (C) the average exon length, (D) the average intron length, and (E) the genome average GC content. Black lines show the estimated regression coefficient. The PGLS r2 and associated P value are shown above each plot. The data and code to generate this figure can be found in https://doi.org/10.5281/zenodo.12608914. (TIF) [file pbio.3002733.s003.tif]

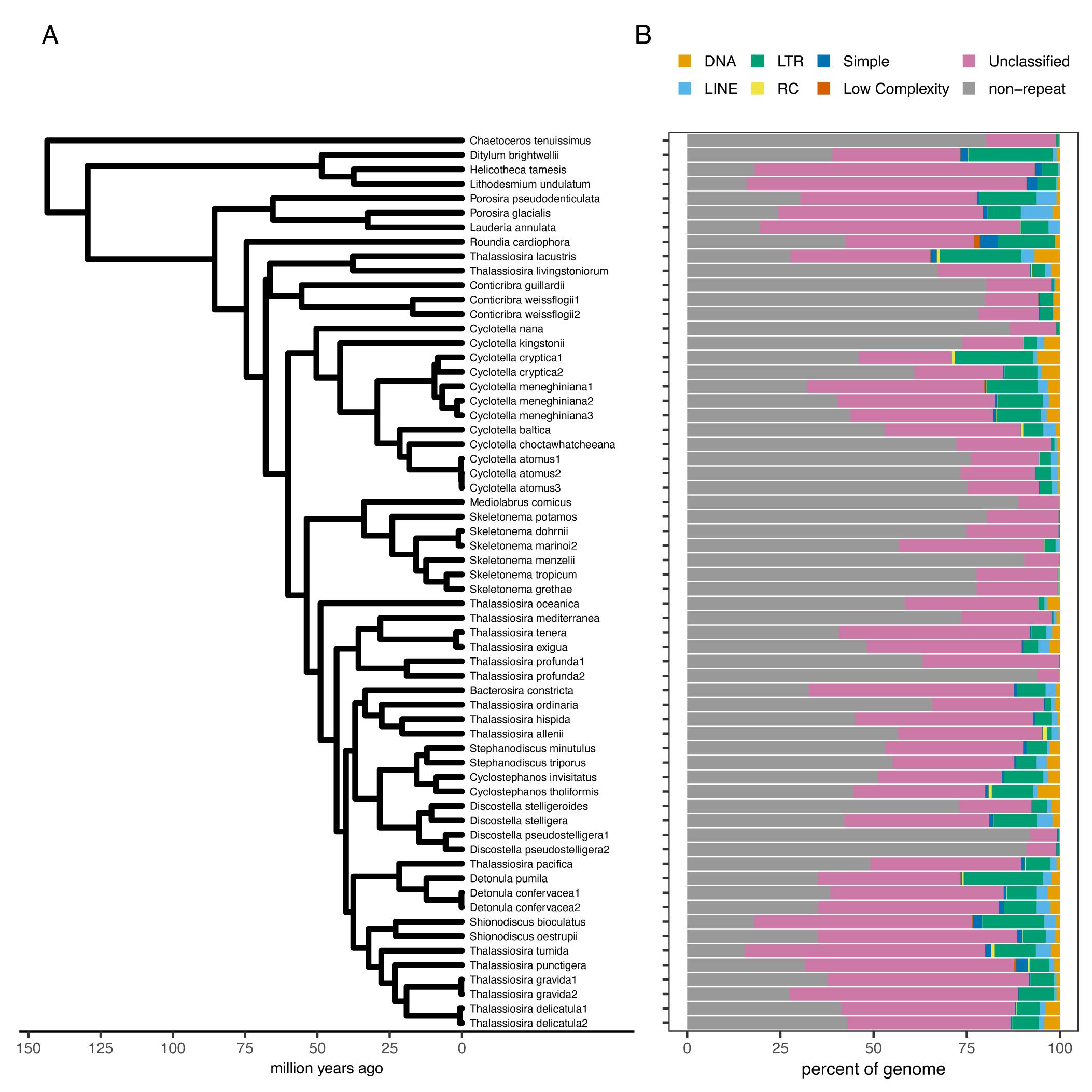

Supplement: S4 Fig — (A) A time-calibrated phylogeny of the diatom order Thalassiosirales, modified from [35]. Strain numbers follow the species names. (B) Stacked bar plot showing the percentage of each genome belonging to different repetitive element classes as estimated using dnaPipeTE. Colors denote the different repeat classes and gray represents the percentage of the genome that is non-repetitive. Abbreviations: DNA, DNA transposons; LINE, long interspersed nuclear elements; LTR, long terminal retroelements; RC, rolling circles or Helitrons. The data and code to generate this figure can be found in https://doi.org/10.5281/zenodo.12608914. (TIF) [file pbio.3002733.s004.tif]

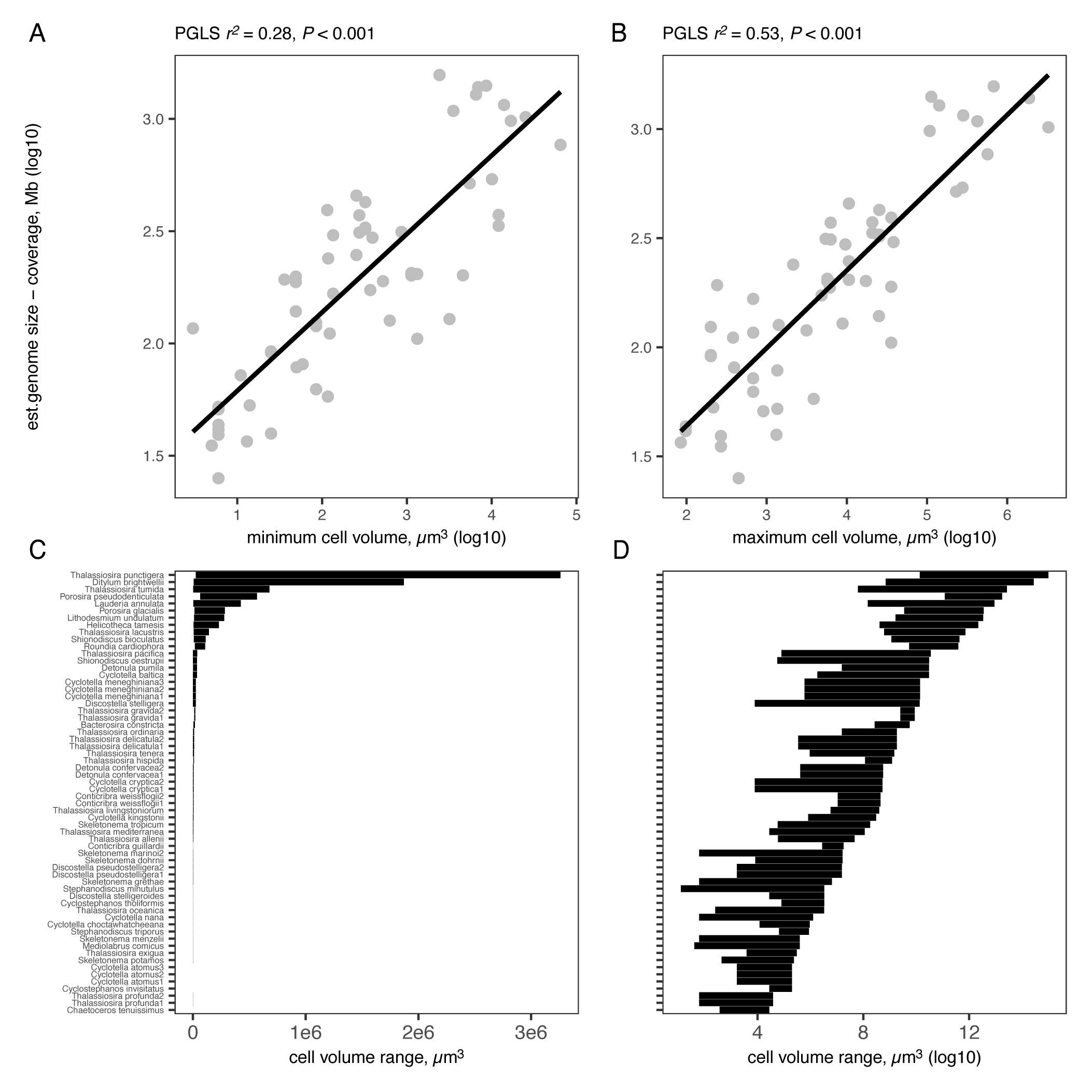

Supplement: S5 Fig — Phylogenetic generalized least squares (PGLS) models predicting genome size by the (A) minimum and (B) maximum calculated cell volume. Black lines show the estimated regression coefficient. The PGLS r2 and associated P value are shown above each plot. The range of cell volume sizes (minimum to maximum) are shown for each species on (C) linear and (D) log10-transformed scales. The data and code to generate this figure can be found in https://doi.org/10.5281/zenodo.12608914. (TIF) [file pbio.3002733.s005.tif]

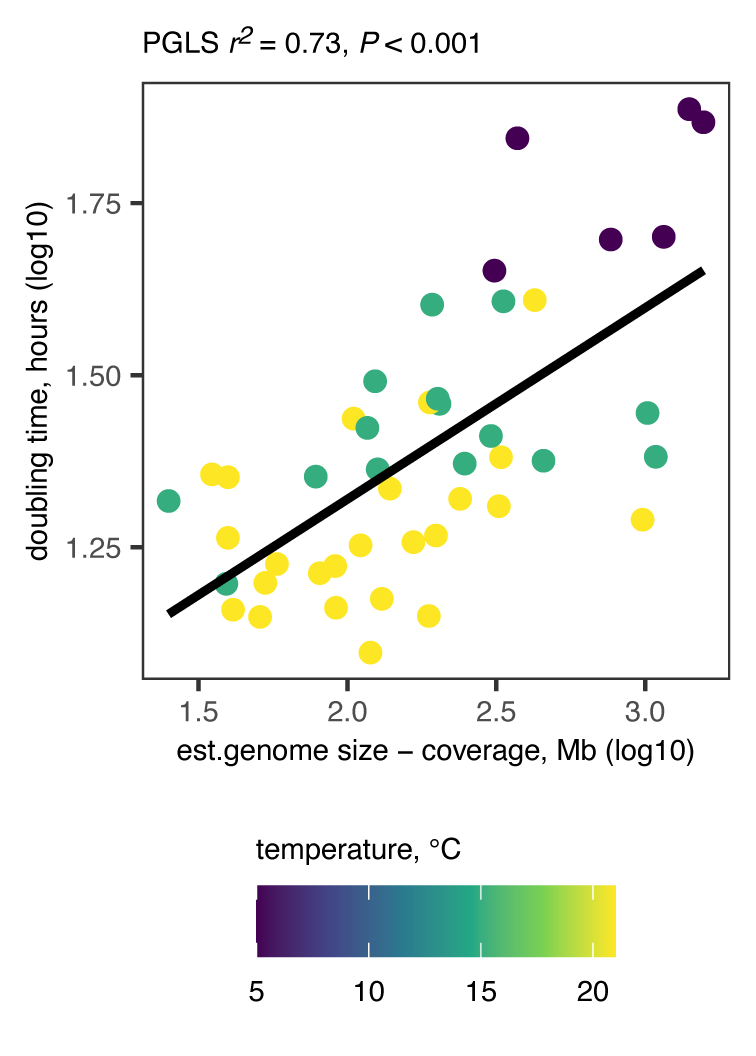

Supplement: S6 Fig — Phylogenetic generalized least squares (PGLS) model predicting doubling time by the additive effects of genome size and temperature. The black line shows the estimated regression coefficient. The points are colored according to the growth temperature of the strain. The PGLS r2 and associated P value are shown above each plot. The data and code to generate this figure can be found in https://doi.org/10.5281/zenodo.12608914. (TIF) [file pbio.3002733.s006.tif]

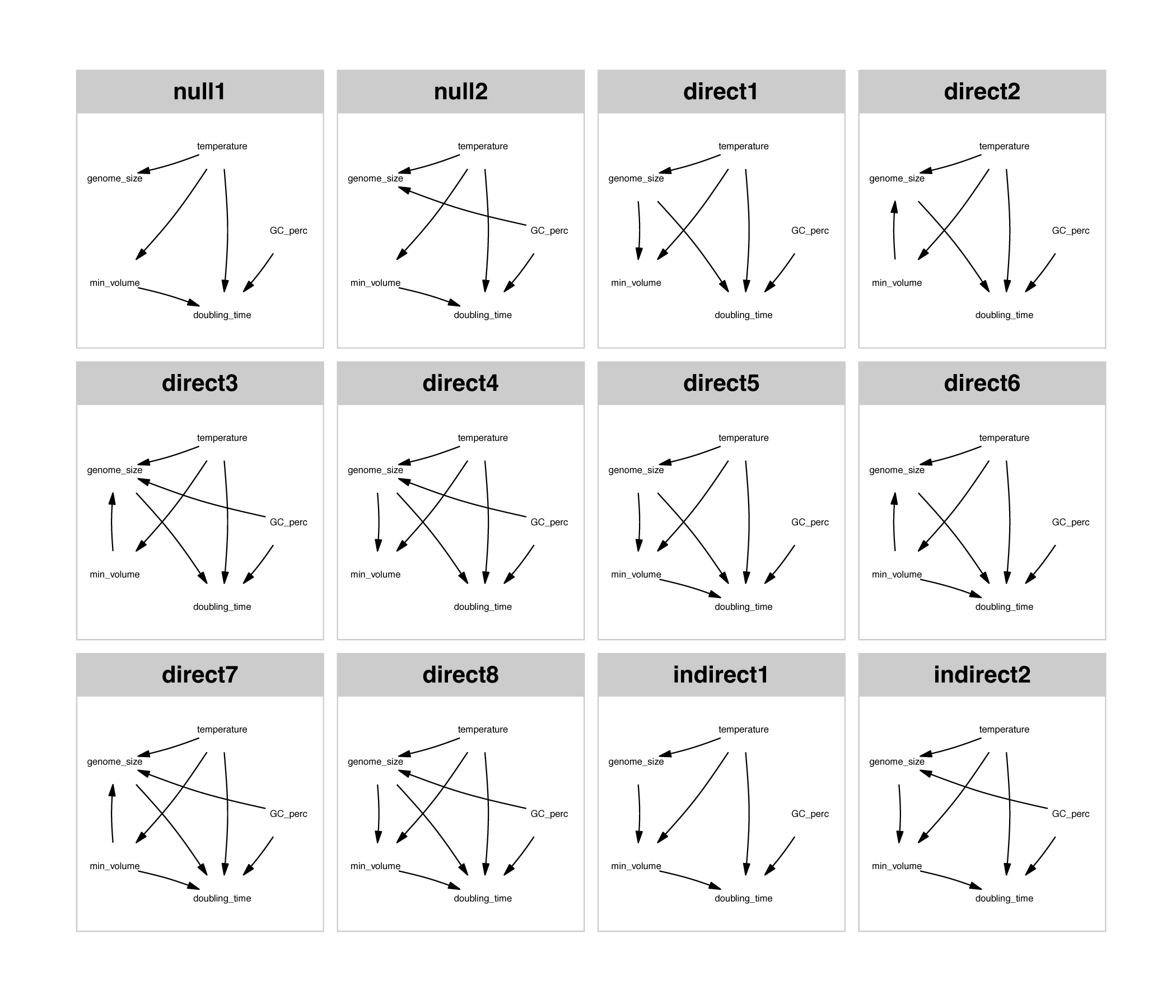

Supplement: S7 Fig — Directed acyclic graphs showing the 14 models tested in the phylogenetic path analysis. Models were defined to test if genome size had no effect (null models), direct effects (direct models), or indirect effects (indirect models) on doubling time. Temperature, GC percentage, and minimum cell volume are also included as variables in the models. Arrow direction indicates the causal relationship being tested between 2 variables. The data and code to generate this figure can be found in https://doi.org/10.5281/zenodo.12608914. (TIF) [file pbio.3002733.s007.tif]

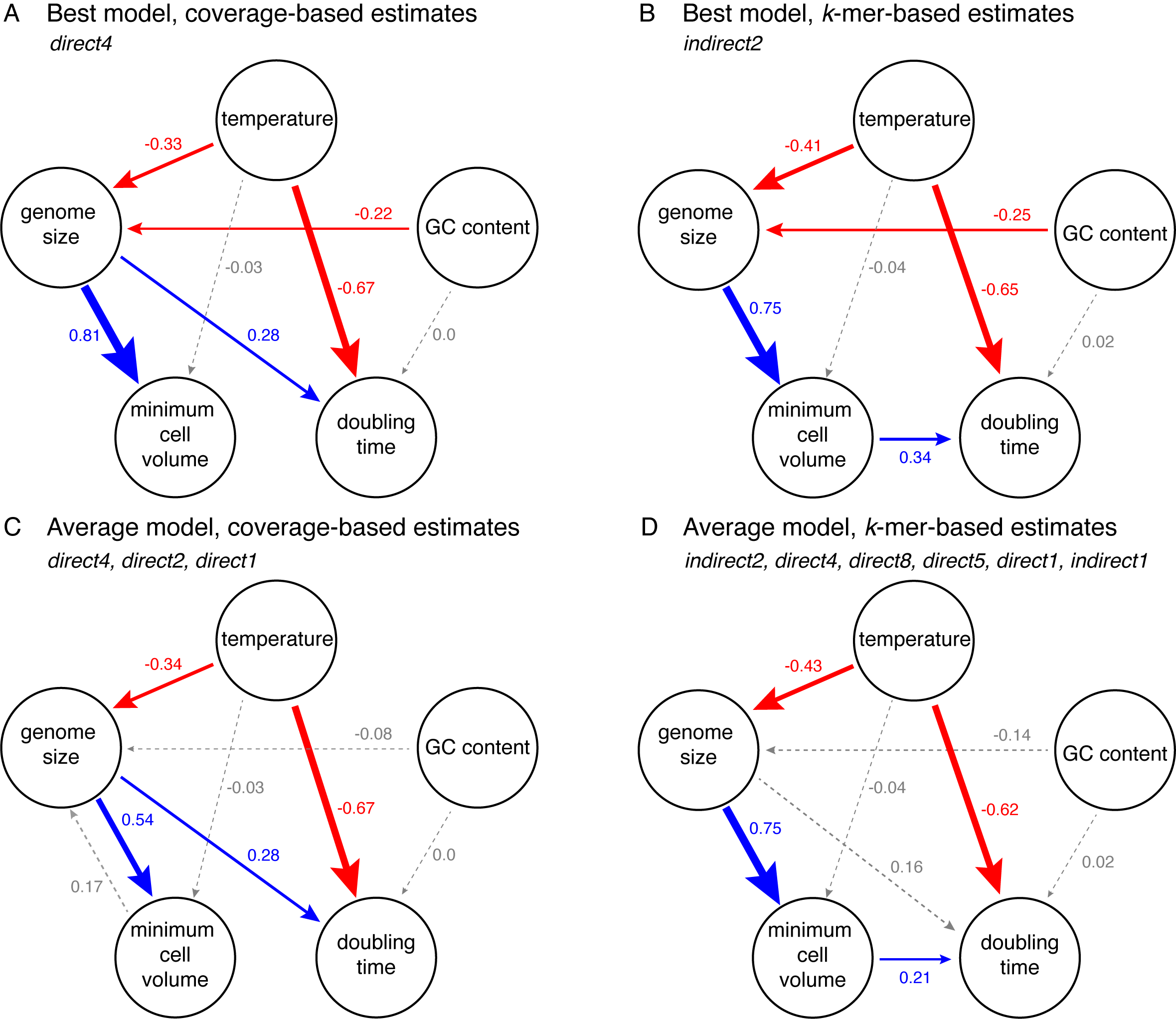

Supplement: S8 Fig — Results of the phylogenetic path analysis. (A, B) Best models and (C, D) averaged models using coverage- (A, C) or k-mer-based (B, D) genome size estimates. Arrow color and width represent the direction and magnitude of regression coefficients, indicated by numeric labels (positive: blue; negative: red; nonsignificant: gray). Full lines show coefficients that differ significantly from 0, whereas negative lines overlap with 0 and are nonsignificant. The data and code to generate this figure can be found in https://doi.org/10.5281/zenodo.12608914. (TIF) [file pbio.3002733.s008.tif]

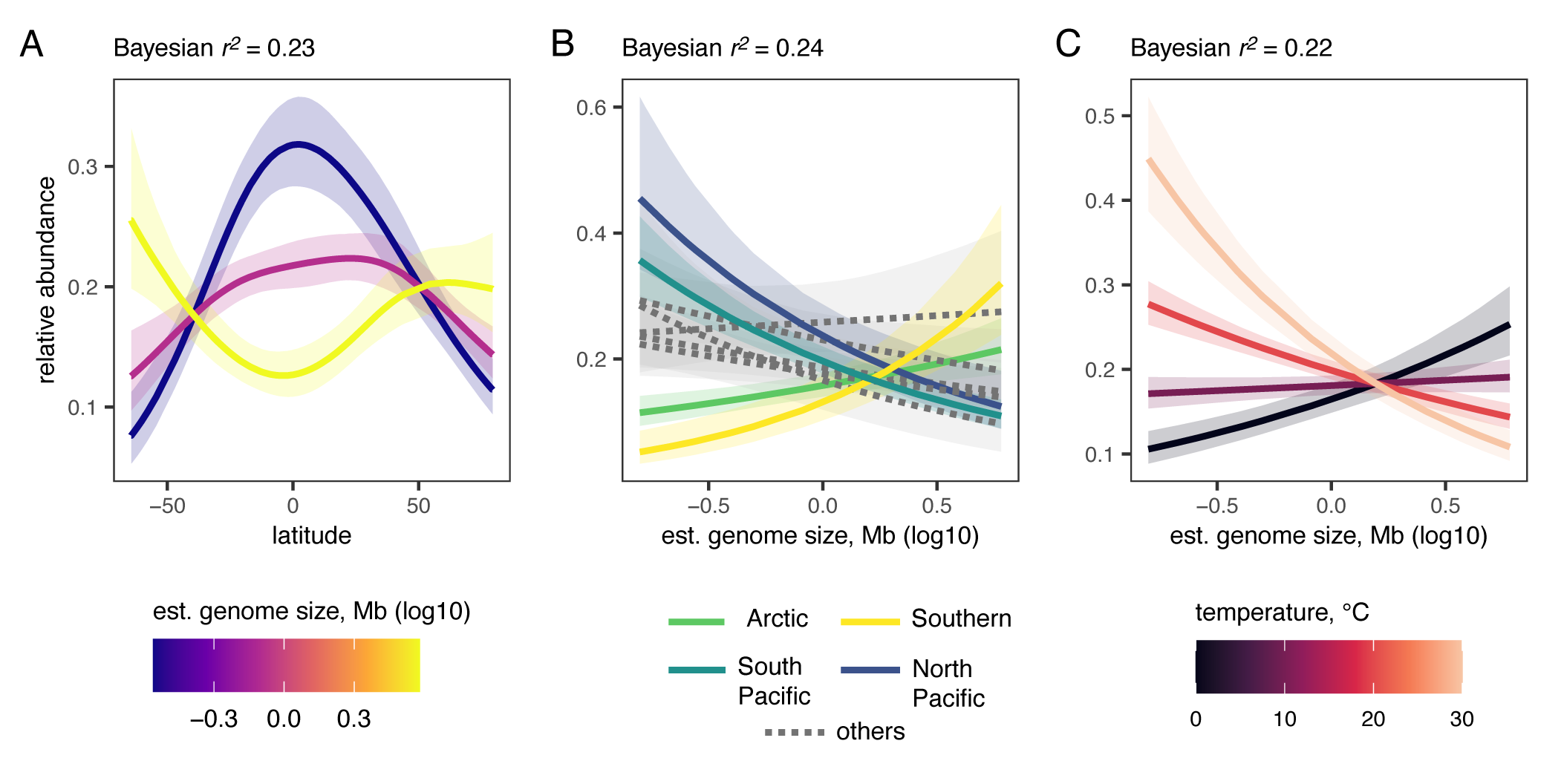

Supplement: S9 Fig — Results from alternative taxonomic assignment of barcodes for 22 diatom species across 210 sampling stations from the Tara Oceans expedition. Panels show Bayesian multilevel regression models predicting relative abundance by the interaction of genome size with (A) latitude, (B) ocean region, or (C) temperature. Nonlinear effects of latitude were modeled in (A) using a generalized additive model. Significant estimates for the Arctic, Southern, South Pacific, and North Pacific Oceans are shown with solid lines in (B). Nonsignificant estimates for the other ocean regions are shown with dotted lines in (B). Although all predictors are treated as continuous in (C), we used the model to predict the interactive effect of 4 discrete temperatures (0, 10, 20, and 30°C) with genome size on relative species abundance. The Bayesian r2 for each model is indicated above each panel. The data and code to generate this figure can be found in https://doi.org/10.5281/zenodo.12608914. (TIF) [file pbio.3002733.s009.tif]
