# Supplementary material for: Diatom abundance in the polar oceans is predicted by genome size
Source: PLoS Biol. 2024 Aug 8;22(8):e3002733. doi: 10.1371/journal.pbio.3002733 (PMC11309476; doi:10.1371/journal.pbio.3002733)
Supplement: Attachment — Submitted filename: Reviewer-response-R2.docx [file pbio.3002733.s015.docx]

REVIEWERS' COMMENTS:

Reviewer #1:

[identifies himself as Adam B. Roddy]

This manuscript assesses the relationships between genome size, genome structure, cell size, population growth rate, relative abundance among a group of diatoms by combining laboratory experiments, field surveys, sequencing, and phylogenetic and path analyses. I particularly like the breadth of these approaches that are presented succinctly in one paper. Indeed, it is rare to see a paper that tackles all of these issues at once, and I think that this paper did this very well on the whole.

The paper was also well-written and easy to follow (except see below for suggestions to setup the results and discussion better). However, I do discuss below a few issues that I found concerning.

First (and easily addressable) is that the manuscript cites very little previous literature (particularly in the introduction), including highly relevant literature. In one Google Scholar search, I found a paper about temperature effects on cell size in diatoms and its implications for Bergmann's rule, but this paper was not cited in this manuscript. I find it odd to have missed such basic information. Furthermore throughout the manuscript, from the very beginning of the introduction to the very end of the discussion, there are very broad statements made that lack citation. Stating a generally accepted principle is one thing, but saying something like "Theoretical studies have developed" or "Another equally large body of literature has sought" without providing a single citation to even a review paper is odd.

Response: The revised manuscript includes additional references.

Second, I think the logic describing presumably causal relationships is confused and muddled throughout the manuscript. (I reference some of these below.) For example, having a larger cell does not mean that there must necessarily be a larger genome. Nor does having a larger genome necessarily that a mature cell will be larger. Rather, genome size determines the minimum cell size, and maximum mature cell size would determine the maximum possible genome size.

Response: We agree with this point. As we describe in more detail below, diatoms have clear and well-documented size ranges. They don’t grow into mature sizes the way plant or animal cells do. For the reason stated here by the reviewer, our analyses used minimum cell size. It is worth noting that analyses with maximum cell size give similar results (S5 Fig).

Moving beyond thing about regression relationships (which show a line through the mean of the population) and thinking more about the outer limits of the point clouds would be useful. To be clear, I don't think that the way these ideas are discussed is any worse in this manuscript than in most other papers. And I am happy that this manuscript performs a path analysis, even if this analysis is based on mature cell size or mean traits.

Response: As discussed in more detail below, our analysis is not based on mature cell size or mean trait values, but rather genome size and minimum cell size.

(Our statistics are fundamentally limited to address the effects of genome size on cell size because our methods predominantly are focused on elucidating mean effects and not the limits of data. This is why I argue below for having clear inductive reasoning that is independent of statistical results [which are inherently deductive].)

Response: If we understand this comment correctly, by conducting the same analysis on minimum cell size and maximum cell size separately (see S5 Figure), our analyses are based on the limits of the data, not the mean values, as they pertain to cell size.

pg 3 para 1: These references to the literature could be better supported by, well, references to the literature. This is particularly notable given that only 5 papers are cited in the entire Introduction section.

Response: The revised manuscript includes additional references.

Diatoms are outside my normal field, but I do find strange how little reference to prior work there is in this manuscript. For example, Adams et al. (2013 GCB, "Diatoms can be an important exception to temperature-size ruels at species and community levels of organization") is not cited, even though it uses a quasi-experimental approach to test whether temperature impacts diatom cell size, and, by extension, whether diatoms may follow Bergmann's Rule. (Notably, they do not sample across latitudes.) Thus, while this manuscript is easy-to-read and clearly written, I am concerned that important prior literature has been ignored.

Response: The revised manuscript includes reference to Adams et al. (2013), which looked at communities and several dozen individual species across geothermal streams in a relatively confined area in Iceland:

“Finally, a field study of freshwater benthic diatoms from geothermally heated streams found no evidence for Bergmann’s rule (Adams et al. 2013), suggesting possible differences in diatom size–abundance relationships across ecosystem types or phylogenetic lineages.”

These are assemblages of freshwater benthic pennate diatoms, so essentially “opposite” in habitat and distant in the phylogeny from our focal group. Given the close proximity of the study sites and taxonomic overlap in the diatom assemblages in the streams studied by Adams et al., there is probably gene flow among sites, which may be one explanation for not finding larger-celled populations (of the same species) in cooler sites.

pg 6, 3 lines up from bottom: is there an extra "top" in this sentence?

Response: Fixed, thank you.

pg 7, 8 lines up: there is an extra "either" in this sentence

Response: Fixed, thank you.

pg 8, bottom: Thsi description of the relationship between genome size and cell size is simplistic. First, while most studies (including the present one) typically present correlations between genome size and mature cell size, there is ample evidence suggesting there is a mechanistic relationship.

Response: It seems that there is a misunderstanding of what measurements we used for our analyses. Our study does not present a correlation between genome size and mature cell size, which is not a measurement that is applicable to diatoms in the same way it is to organisms whose cells grow and expand. Because diatoms have a rigid, silicon-based cell wall, there are clearly defined and easily measured minimum and maximum cell sizes for each species. We conducted two separate analyses: one based on minimum cell volume and one based on maximum cell volume. We state this in the caption for (D) in Fig 1, but we changed our axis label to specify “minimum”. In Fig. 2, our model specifies “minimum cell volume”. S5 Figure compares the results that used minimum vs. maximum cell volume. S8 Figure shows alternative path analyses with minimum cell volume labeled.

Specifically, genome size (by extension, nuclear volume) determines the minimum possible cell size. However, mature cells can be larger than this minimum cell size. In plants, for example, genome size is an exceptionally strong predictor of meristematic cell volume, but only a weak predictor mature cell volume...precisely because mature cells can be larger than their minimum cell size. Thus, genome size does act as a hard, physical limit on cell size, but only on minimum cell size.

Response: The recent review article by Doyle and Coate (2019. Int. J. Plant Sci 180:1-52) takes a much more nuanced view of the effect of genome size on both nuclear volume and cell volume, citing studies in plants that support a greater effect of cell volume on nuclear volume. However, as discussed throughout our responses here, diatom cell size is very different than plants in that there is no tissue differentiation, development, or traditional cell growth, so what the reviewer has interpreted as “simplistic” is our attempt to balance the comparative vast literature about genome size in multicellular organisms with the limits of how processes in other organisms translate to the diatom system studied here.

Cell size in diatoms is fundamentally different from most other organisms, which makes the diatom system a strong one for exploring the main questions in our study. Diatom cells do not grow/expand in the same way plant or animal cells do. The diatoms analyzed in this study are cylindrical in shape and encased in a rigid glass cell wall that, by its nature, does not grow in the traditional sense because it does expand from an immature to mature size the way plant cells do. We described it this way in the manuscript:

“In addition to the ecological consequences, our results highlight the unique cellular tradeoffs imposed by changes in genome size in diatoms. Diatoms reproduce asexually throughout most of their life history and are unusual in that one of the two daughter cells following a mitotic event is smaller than the parent, leading to a reduction in the average diameter of a cell lineage over time, eventually triggering sexual reproduction and restoring the maximum cell size [37].”

One strength of the diatom system is that there is abundant data on the natural cell size range for most diatoms, including all the ones in our study. We compiled these measurements from the literature and from our own measurements of cultured cells, which over time and across successive cell divisions, express the full size range. We analyzed the data using both minimum and maximum cell volumes (S5 Fig). Because genome size does, as the reviewer states, probably impose a physical limit on minimum cell volume, all of the analyses presented in the paper are based on minimum cell volumes. The review by Doyle and Coate (2019) expresses less confidence in this relationship between genome, nuclear and cell size. We welcome these debates and discussions because, in our view, it highlights the relevance of our work.

We modified our text in several places throughout to try to strike the balance requested by the reviewer that is true to the literature and to the realities of our system.

It might be possible to estimate the minimum cell size from genome size for these diatoms. How close mature cell sizes are siting to this minimum cell size bound likely reflects the how strongly selection on cell size would impact minimum cell size and thus genome size.

Response: The data and analyses presented in the paper are based on minimum cell volumes, i.e., cell volume at the lowest end of their size range. We have accurate, replicate estimates of minimum cell volume for each species. As stated above, the caption for (D) in Fig 1 specifies minimum cell volume, but we changed our axis label to specify “minimum” also. In Fig. 2, our model specifies “minimum cell volume”. S5 Figure compares the results that used minimum vs. maximum cell volume. S8 Figure shows alternative path analyses with minimum cell volume labeled.

I am not an expert on diatoms, and my thinking is based on plants. But, even if there are no changes in genome size, cell size can change (because mature cells are larger than minimum cell size)-and this variation in mature cell size CAN be adaptive.

Response: Since diatoms do not grow/expand to maturity in the same way plants do, we were able to analyze genome size as it relates to known minimum and maximum cell volumes. Analyses based on minimum cell volume are presented in the main manuscript, and analyses based on maximum cell volume are shown in the supplemental results (S5 Figure).

It's also worth noting that because the effects of genome size on nucleus size are extremely tightly linked physically, (on my opinion) the only relevant point about adaptation in this relationship is that nucleus size remains just a bit larger than the genome instead of growing much larger than the genome and being tied more to fluctuations in cell size.

It's vitally important when talking about genome size, nuclear volume, and cell size to be very specific and clear about the causal/mechanistic relationships. This is why correlations (such as those presented in Figure 1 and in most other papers) are merely correlations and do not necessarily describe any physical relationship between genome size and cell size. Even if phylogenetic regressions between these variables are statistically significant, that relationship still does not say anything about the physical relationship between the two traits; rather, it is about how the coevolve. And trait coevolution could, in theory, be driven largely by a physical mechanism or result from selection acting on both traits. Inductive reasoning-and not only statistical relationships-would be required to describe the causal relationships.

Response: Thank you for pointing this out. In response to this and other comments above, we tried to be more clear about the nature of the possible relationship between genome, nuclear, and cell size in diatoms.

pg 9, line 3: When I read "laboratory growth experiments", I was at first confused. Admittedly, I had not yet read the methods since they are at the end. I suspect that the methods will be at the end in the final version of the paper. In that case, I think it would be worth either in the Introduction or in the Results giving a little shout-out or sign-posting so that the reader knows what data are being used. I had assumed that growth rate data were compiled from the literature, so clarifying it was measured would help.

Response: We have now clarified in the Results section that the growth rates were directly estimated in the lab:

“We measured maximum growth rates for the species in our study (S1 Table) to test whether genome size is a predictor of cell division rate and found that species with larger genomes did indeed have longer doubling times (i.e., slower growth rates) (PGLS r2 = 0.42, P < 0.001) (S6 Fig).”

pg 9, para 2 line 1: This sentence sets up a false dichotomy. By definition, what makes genomes bigger is having more nucleotides. A larger cell is not a mechanistic explanation for larger genomes. Rather, it is a correlation, or a result of having a larger genome, but a larger cell per se cannot tell us anything about the size of the genome EXCEPT the maximum size of the genome (i.e. the genome cannot be, by definition, larger than the entire cell).

Response: The purpose of this sentence is simply to say that although previous flow cytometry studies found correlations between genome and cell size, DNA sequencing allowed us to go further and show that genome size was driven by repetitive DNA.

pg 9, last para: I find the logic here confusing. It is a strange argument to say that genome size is more important than cell size to an organism's ecology. Does genome size itself limit rates of diffusion into the cell? Does genome size per se determine how many cells can be packed into a space? The answer to both questions is no. What CAN be said is that genome size is a stronger correlate of ecology than cell size. But that is a different point: in our subjective position of trying to characterize an organism's ecology, genome size is very useful...in contrast to saying that the diatom's way of life is more defined by the size of the genome than by the size of its body.

Response: We changed the topic sentence to state: “Although cell size is often considered a ‘master’ phytoplankton trait, our results highlight the ecological importance of genome size as well.” We are then careful not to not minimize the ecological importance of cell size.

pg 19 top: Evidence supporting Lynch's ideas about Ne and genome size is severely lacking. Multiple papers in other systems (fishes, mammals) have tested for a relationship between Ne and population size and find no relationship.

Response: The evidence for this hypothesis is mixed, with some datasets supporting it and others not. We have added this caveat to our manuscript with citations to relevant literature:

“Although evidence for this hypothesis is mixed, [54–56], the inclusion of population genetic parameters in our models might have shown whether diatoms with smaller effective population sizes are potentially more susceptible to non-adaptive genome expansions due to genetic drift [53].”

pg 11 middle: This paragraph implies a positive correlation between genome size and abundance in vertebrates (based only on salamanders). Doesn't that contradict the relationship reported in this paper (restricting to the black points from the lower latitudes where salamanders are also predominant)?

Response: We have added the caveat that this relationship is not related to a temperature gradient, but there does seem to be a genome-size/abundance relationship in salamanders: “Although not related to a temperature gradient, salamanders are among the most abundant animal groups in many terrestrial ecosystems [64], and they have among the largest known genomes in the vertebrate lineage [65].”

pg 11, last line: "nucleus size is positively correlated with genome size across the tree of life"...where is the citation to this statement?

Response: Citations added.

I hope the authors have found these comments constructive and useful. I commend them for what is, on the whole, a very good paper.

Response: Thank you for taking time to read and provide constructive comments on our manuscript.

Sincerely,

Adam B. Roddy

Reviewer #2:

This manuscript presents analyses of an impressive number of novel diatom assemblies (53).

The authors analyze 53 original genome assemblies' sequences and 14 previously published assemblies of 51 species of diatoms from the Thalassiosirales order. They use this dataset to investigate the relationship between genome size, different genomic traits, growth rates, and the relative abundance of diatoms in the ocean.

They report a positive linear relationship between genome size and abundance in the Arctic and Southern Oceans (Figure 3D), whereas all other regions either had no or a negative effect of genome size on species abundance.

It seems very misleading to me that the title and abstract of this manuscript suggest that genome size predicts Thalassiosirales abundance in the ocean.

Response: We have modified the manuscript title and abstract to better reflect that genome size predicts abundance in polar oceans.

Major comments

1.	Please estimate the significance of the relationships between genome size and relative abundance of species in Figure 3C. It is likely there is no

Response: We have now noted in the text and Figure 3 where the relationship between genome size and relative abundance are significant. Each of the three models (including the alternate OTU assignment shown in Figure S9) has a significant interaction between genome size and relative abundance. Since we built Bayesian models, there is not a traditional P value for assessing significance. Instead, if the mean and 95% confidence intervals of the estimated effects do not overlap with 0, they are considered significant. We have clarified this interpretation in the Methods as well.

Significant interactions in Figure 3 and S9 are now indicated using either solid (significant) or dashed (non-significant) lines for each model.

2.	P5. "there was no association between genome size … with the presence of polyploidy". The authors seem to confuse the actual genome size of a species (that would take ploidy level into account) and the haploid genome size. The latter can be estimated from an assembly when the two parenral chromosome copies are not too polymorphic. Please clarify.

Response: We are referring in all cases to haploid genome size, and we have now clarified that throughout the Results section of the revised manuscript. Our estimates of ploidy level are described in the “Ploidy estimation” section of the Methods, and the ploidy predictions (all diploid) are presented in S1 Table. All species are predicted to be diploid.

3.	P19. It is not clear to me how the relative abundance of each species was estimated from the Tara dataset. Usually, relative read coverage is taken as a proxy of relative abundance, this is however not clear from this paragraph. Also, taking 18SrDNA as a proxy of abundance of different species has many biases. The main bias is that it will overestimate the presence of species with a larger copy numbers of the 18SrDNA operon, and thus probably the species with larger genome sizes. I would recommend using a single copy gene (i.e. BUSCO genes) to estimate relative species abundance from metagenomic data.

Response: We used previously assembled and clustered V9-18S rRNA OTU sequences from the Tara Oceans expedition that were made publicly available from Zenodo and provided as a supplement to Ibarbalz et al. 2019 (<https://doi.org/10.1016/j.cell.2019.10.008>). Read counts from that study for each OTU were used as the abundance estimate. We have clarified throughout the text that these are relative abundances and clarified how they were calculated in the Methods.

We agree that there are caveats in 18S-rRNA metabarcoding due to copy number variation across species and lineages. However, Malviya et al. 2016 PNAS (<https://doi.org/10.1073/pnas.1509523113>), in a study focused on diatom diversity and abundance in the Tara Oceans dataset, found general congruence between molecular and morphological estimates of abundance. They concluded that copy number variation does not appear to be a major concern for diatoms. Further work by Martin et al. 2022 (<https://doi.org/10.3897/mbmg.6.85794>) compared microscopy and molecular estimates of diatom diversity before and after applying a correction factor based on biovolume. They found that the morphology and molecular metabarcoding abundances largely agreed in diatoms, and that the application of the correction factor underestimated actual diatom abundance. Additionally, Karlusich et al. 2023 Mol Ecol (<https://doi.org/10.1111/1755-0998.13592>) compared the V9-18S abundances from Tara Oceans with microscopy and the putatively single-copy gene psbO, concluding that diatom abundances were robust across these different measurements even in different size fractions.

Regarding single copy BUSCO genes, very few genes exist as a single copy in the genome, and copy number can vary across species even for very-low-copy-number genes. If there were a single-copy BUSCO in diatoms, it would have to evolve quickly enough to discriminate among species in the same V9-18S sequences can. Recent work by Karlusich et al. (2023) (<https://doi.org/10.1111/1755-0998.13592>) identified the putatively single-copy, nuclear-encoded, plastid targeted gene psbO as a potential marker for estimated abundance from metagenomes. The raw metagenome shotgun sequencing data produced by the Tara Oceans and Tara Arctic expeditions are available from the European Nucleotide Archive (Accessions PRJEB4352 and PRJEB9691). Just these two accessions contain a total of 2390 raw shotgun sequencing files whose combined compressed size is 27 TB. We are not currently positioned to download, store, and analyze this dataset. These are raw reads, so each of the 2390 files would need to be quality checked, trimmed, and aligned to a set of reference sequences to generate the requested abundance estimates. Identifying psbO as a candidate marker was the subject of an entire paper, and verifying whether this candidate gene would work for these diatoms, while potentially useful, is beyond the scope of this paper, especially considering the many studies cited above showing that V9-18S works well for estimating diatom abundance.

Minor comments

1.	There are 116 genome assemblies of Bacillariophyta available in Genbank, I was wondering why the dataset was restricted to Thalassiosirales and did not include well known genomes such as Phaeodactylum and Fragillaropsis genomes.

Response: Over half of the 116 diatom genomes on GenBank were sequenced in our lab, mostly for the present study. Phylogenetic comparative methods perform best with dense and even sampling across a clade, which is what we have done for Thalassiosirales. We chose this clade in part because they are planktonic and abundant across the global ocean, including in polar and temperate regions. Focus on a planktonic lineage allowed us to use the Tara Oceans database, which focused exclusively on the plankton. Many of the genomes on GenBank are from habitats not sampled by the Tara expedition (e.g., the benthic or tychoplanktonic habitats of Phaeodactylum, Cylindrotheca, Nitzschia). Finally, some of the other diatom genomes on GenBank do not have associated short reads, which are necessary to estimate genome size, since assembly length is not always an accurate predictor of genome size due to potential incompleteness.

2.	The genome size is estimated by two methods : from kmer prevalence and coverage mode. The accuracy of these two genome size estimations as compared to the actual genome size (e.g. including ploidy levels) should be discussed in more detail.

Response: The predicted ploidy levels are presented in S1 table. All species are predicted diploids. Most genome size estimates we provide are the first estimates for these species, therefore we cannot compare these estimates to acknowledged genome size. Bioinformatic approaches to genome size estimation have been demonstrated to be as accurate as experimental approaches in many different species (<https://doi.org/10.1534/g3.120.401028>, <https://doi.org/10.1111/1755-0998.13570>). The accuracy of k-mer approaches depends upon having sequencing coverage >10x, but using read coverage allows the accurate estimation of genome size down to 1x coverage.

Comparison of our estimates to previous flow cytometry estimates (von Dassow et al. 2008) provide similar haploid genome sizes for three strains:

|  | Our estimates | Von Dassow et al. 2008 |
| --- | --- | --- |
| Cyclotella nana CCMP1335 | 33 Mb | 36 Mb |
| Thalassiosira oceanica CCMP1005 | 76 Mb | 79 Mb |
| Conticribra guillardii CCMP988 | 133 Mb | 154 Mb |

We have included this comparison in the Results section:

“Our estimates of genome size were similar for the three strains in our dataset with genome size estimates from flow cytometry (S1 Table). For example, our estimate for Cyclotella nana CCMP1335 was 33 Mb, while flow cytometry estimated it at 36 Mb (S1 Table).”

Reviewer #3:

The manuscript presents a thorough analysis of the relationships between cell size, genome size and population abundance in marine diatoms. The authors use newly generated and existing data on diatom cell size, genome size and composition, phylogenetic relationships and abundance in the world oceans to see if genome size is a good predictor of cell size, doubling time and species abundance in the environment.

I read the manuscript with great interest, as the authors ask an important question of how the important traits in protists are correlated and whether they can be used to predict species abundance. They use a variety of appropriate methods and datasets to address it. This is a well-conceived and expertly executed project that provides a wealth of novel results; I do not have any major concerns about this manuscript. I only have a couple of suggestions for the path analysis and the discussion.

Response: Thank you for this nice comment.

I was especially intrigued by the team's investigation of whether the genome size influences cell size or vice versa. It was quite surprising to see that the results of their path analysis suggest that genome size determines the cell size in marine diatoms. I would expect the opposite causal relationship, where cell size influences genome size. For example, it is well known that under low nutrient conditions there is a selection towards smaller cell sizes. This mechanistic explanation is well accepted; but thinking about the selection mechanisms acting directly on genome size is less well developed. Could it be that low nutrient conditions would select for small genomes because of nutrient limitation of the DNA synthesis? It would, therefore, be useful if the authors would discuss possible selection mechanisms determining genome size under different conditions that would in turn influence the cell size.

Response: Figuring out the push and pull relationship between genome size and cell size was one of the main motivations of our study. Different people have different expectations for which is driving which, in part because formal tests of this question are difficult. The dataset we collected, together with the application of path analysis in phylogenetic studies, allowed us to approach this question in a new way.

The point about low nutrient conditions is well taken. We cite Hessen et al. (<https://doi.org/10.1016/j.tree.2009.08.004>), a paper that was highly influential to us in thinking about our results:

“Larger genomes also require additional investments of N and P to replicate and maintain, so in species with large genomes, these two essential nutrients cannot be allocated to RNA, ribosomes, and proteins, reducing growth rates and, over longer timescales, selecting for smaller genomes under conditions of nutrient limitation [41].”

I would also like to suggest using nutrient concentration as a variable in path analysis, along with temperature, if such data are available. Conducting such an analysis could help understand the potential selective pressures on genome size and cell size, as nutrient concentration is a key factor affecting phytoplankton, including diatom, ecology and evolution.

Responnse: Thank you for the suggestion. And we agree that the inclusion of other variables would almost certainly yield a better-fitting model. As currently implemented, multiple measurements for a single variable cannot be incorporated into phylogenetic path analyses, which is also why abundance was not analyzed in the path analysis.

We were, however, interested in the effect of nutrients on our models, so we compiled measurements of nitrate (N), phosphate (P), iron (Fe), and silicate (Si) taken for each Tara Oceans sample included in our analysis. As stated above, phylogenetic path analysis does not allow multiple measurements per species, therefore we cannot employ nutrient measurements into that method. We instead used a predictive model with all the suggested variables. Therefore, we took the existing abundance models we built and added in additional interactions between genome size (GS) with each of N, P, Si, and Fe:

Abundance ~ GS*temperature + GS*N + GS*P + GS*Si + GS*Fe

Abundance ~ GS*region + GS*N + GS*P + GS*Si + GS*Fe

Abundance ~ GS*latitude + GS*N + GS*P + GS*Si + GS*Fe

In all models, nitrate had a significant effect on overall abundance and only had a significant interactive effect with genome size in the smaller OTU dataset when temperature was an added variable. This would suggest that N is important for overall species abundance but makes very little difference on the abundance of larger or smaller species. Phosphate never had a significant effect on species abundances in any model. Only one model had a significant interaction between iron and genome size, in the smaller OTU dataset when latitude was a predictor. Silicate had an effect (with or without interaction on genome size) in some models, showing, unsurprisingly, that these species are more abundant when silica availability is higher.

Importantly, in all these models that included nutrient variables, the interactions of temperature, ocean region, and latitude with genome size all remained significant predictors of abundance.

To do this properly, we would probably need to incorporate other variables, such as seasonality, into our models, which increases the complexity in a lot of ways and begins drifting from our original research questions. Our hope is that true phytoplankton ecologists who work with these kinds of datasets more than us, especially those focused on trait-based phytoplankton ecology, will incorporate genome size as a trait into their models.

We appreciated this comment and spent a good deal of time looking into it, but we felt in the end that adding nutrients was potentially fraught with difficulty and went beyond the scope of our study.
